# Plug‐and‐Play Centrifuge‐Only Device for Rapid Sepsis Diagnosis

**DOI:** 10.1002/adhm.202503651

**Published:** 2025-09-02

**Authors:** Mohammad Osaid, M. Henar Marino Miguélez, Berke Bayrak, Büsra Betül Özmen‐Capin, Volkan Özenci, Wouter van der Wijngaart

**Affiliations:** ^1^ Micro and Nanosystems KTH Royal Institute of Technology Stockholm 100 44 Sweden; ^2^ Department of Clinical Microbiology Karolinska University Hospital Huddinge 141 86 Sweden

**Keywords:** bacteria, blood, bloodstream infections, diagnostics, maldi‐tof, microfluidic, sepsis

## Abstract

Sepsis is a time‐critical condition causing over 13 million deaths annually, with each hour of treatment delay in patients with septic shock increasing mortality by 8%. Rapid pathogen identification is crucial, yet current workflows depend on multiple culture steps that delay pathogen identification and targeted treatment by days. A plug‐and‐play, fully automated centrifuge tube is presented that isolates and concentrates bacteria directly from blood or blood culture using only conventional lab centrifuges. Each tube can process 7.5 ml of sample and yields, within 40 min, a 0.7 mL clear suspension with greater than threefold enhanced bacteria concentration and > 99.9% blood cell rejection, ready for downstream detection. It is demonstrated that this approach supports key diagnostic workflows, including 1) a novel isolate‐then‐culture strategy detecting bacterial concentrations as low as 10 CFU/mL; 2) direct matrix‐assisted laser desorption ionization time‐of‐flight (MALDI‐TOF) identification, bypassing subculturing, and; 3) microfluidic single‐cell detection. This fully automated platform is compatible with existing centrifuges, is anticipated to facilitate broader adoption in routine clinical practice, while its ability to enable rapid, same‐workshift bacterial enhancement can reduce diagnostic time by about one day in the context of time‐critical sepsis diagnostics.

## Graphical abstract

A fully automated, centrifuge‐only device can isolate and concentrate bacteria from blood for rapid sepsis diagnosis. The device enables >3× bacterial enrichment and removes >99.9% of blood cells. The device supports direct downstream analysis via subculturing, MALDI‐TOF, and microfluidic detection of bacteria.

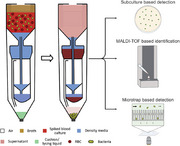

## 1 Introduction

Sepsis is a serious condition where the host's response to infection causes organ damage, and it is responsible for 20% of deaths worldwide.^[^ ^1^, ^2^, ^3^ ^]^ An estimated 25–30% of sepsis cases involve bloodstream infections (BSI).^[^ ^4^, ^5^ ^]^ If left untreated, sepsis can lead to septic shock, where the mortality rate for patients increases by 8% per hour,^[^ ^6^ ^]^ underscoring the critical need for rapid diagnosis in the management of sepsis. Due to the severity of this infection, patients with sepsis often require intensive care unit treatment, which incurs high costs.^[^ ^7^ ^]^ Broad‐spectrum antibiotics are commonly prescribed at the onset of infection. However, this approach is suboptimal; it increases fatality rates^[^ ^8^ ^]^ when it fails and contributes to antimicrobial resistance,^[^ ^9^ ^]^ posing a considerable threat to public health. The state‐of‐the‐art methods for identifying pathogens and performing antimicrobial susceptibility testing (AST) generally take 2–3 days.^[^ ^10^ ^]^ This process is lengthy due to its reliance on multiple culture steps for identification and AST.

In clinical settings, patients' blood samples are first subjected to a blood culture to increase the bacterial count, which typically takes between 4 and 72 h to produce a positive result,^[^ ^11^, ^12^, ^13^ ^]^ with the mean time to positivity being approximately 21 h.^[^ ^14^ ^]^. Once a culture is confirmed positive, it is essential to identify the microorganism and perform AST to prescribe effective antibiotics. However, downstream analyses, such as identification and AST, cannot be performed directly from a positive blood culture because the overwhelming presence of blood cells—on the order of billions per milliliter, interferes with bacterial cells during downstream analysis. The bacterial cells are subcultured on an agar plate for downstream processing, delaying the procedure by another half a day.^[^ ^15^, ^16^ ^]^ Therefore, there is a critical need for powerful and standardized sample preparation methods that can enable downstream analysis directly.

Current methods for identifying bacterial species for BSIs include genotypic techniques (e.g., polymerase chain reaction), phenotypic methods (e.g., subcultures), and mass spectrometry (MS), like matrix‐assisted laser desorbtion ionisation time of flight MS (MALDI‐TOF), which is one of the fastest and most commonly used techniques in well‐equipped clinics.^[^ ^17^ ^]^ However, like other methods, MALDI‐TOF also commonly requires an additional subculturing step before the identification step. Although faster alternatives to subculturing exist, such as differential centrifugation with lysis^[^ ^18^ ^]^ or commercial kits like Sepsityper^[^ ^18^, ^19^ ^]^ and the Accelerate Arc System,^[^ ^20^ ^]^ these methods are more labor‐intensive, involve multiple centrifugation steps, are more expensive, require higher hands‐on time than subculturing, and are highly user‐dependent. Moreover, most of these methods start by mixing a lysing agent with blood or blood culture, which makes the entire approach less suitable for downstream rapid AST, as the lysing agents can slow down bacterial growth.^[^ ^21^, ^22^ ^]^

Traditional AST methods involve growing bacteria in broth or on agar plates in the presence of antibiotics, a time‐consuming process that can take 18–24 h due to reliance on macroscopic bacterial growth identification.^[^ ^23^ ^]^ Alternative microfluidic‐based technologies can perform AST within hours by trapping and imaging single bacterial cells, but they mostly require a pure bacterial culture.^[^ ^24^, ^25^ ^]^ Thus, sample preparation remains a bottleneck for these technologies to function directly from blood or positive blood cultures.

Robust sample preparation that eliminates the need for culture or subculture could significantly accelerate the diagnostic process. Numerous methods and devices have been developed for sample preparation from blood or blood cultures, tailored to different downstream processing methods.^[^ ^17^ ^]^ Common techniques for isolating and concentrating bacterial cells include sedimentation velocity‐based methods,^[^ ^26^, ^27^, ^28^ ^]^ filtration,^[^ ^29^, ^30^ ^]^ magnetic bead capture,^[^ ^31^ ^]^ acoustophoretic techniques,^[^ ^32^ ^]^ inertial and elastoinertial microfluidics,^[^ ^33^, ^34^ ^]^ surface acoustic wave (SAW)‐based approaches,^[^ ^35^ ^]^ and dielectrophoresis.^[^ ^36^ ^]^ While centrifugation and filtration methods offer high throughput and are commonly used in clinics for sample preparation for MALDI‐TOF MS or AST,^[^ ^37^, ^38^ ^]^ they require multiple steps for downstream processing, making them labor‐intensive, susceptible to contamination, highly user‐dependent, and often less accurate in the case of MALDI‐TOF MS.^[^ ^18^ ^]^ Other methods suffer from low throughput or are too complex for clinical application.

This study developed a high‐throughput, non‐selective, and fully automated device for sample preparation that is easily integrable into existing diagnostic pipelines, enabling direct analysis without solid culturing steps for methods such as MALDI‐TOF MS, single‐cell detection, and rapid AST. It also standardizes the entire sample preparation process, improving consistency and reliability.

## 2 Results

### 2.1 Device Design

**Figure 1:**
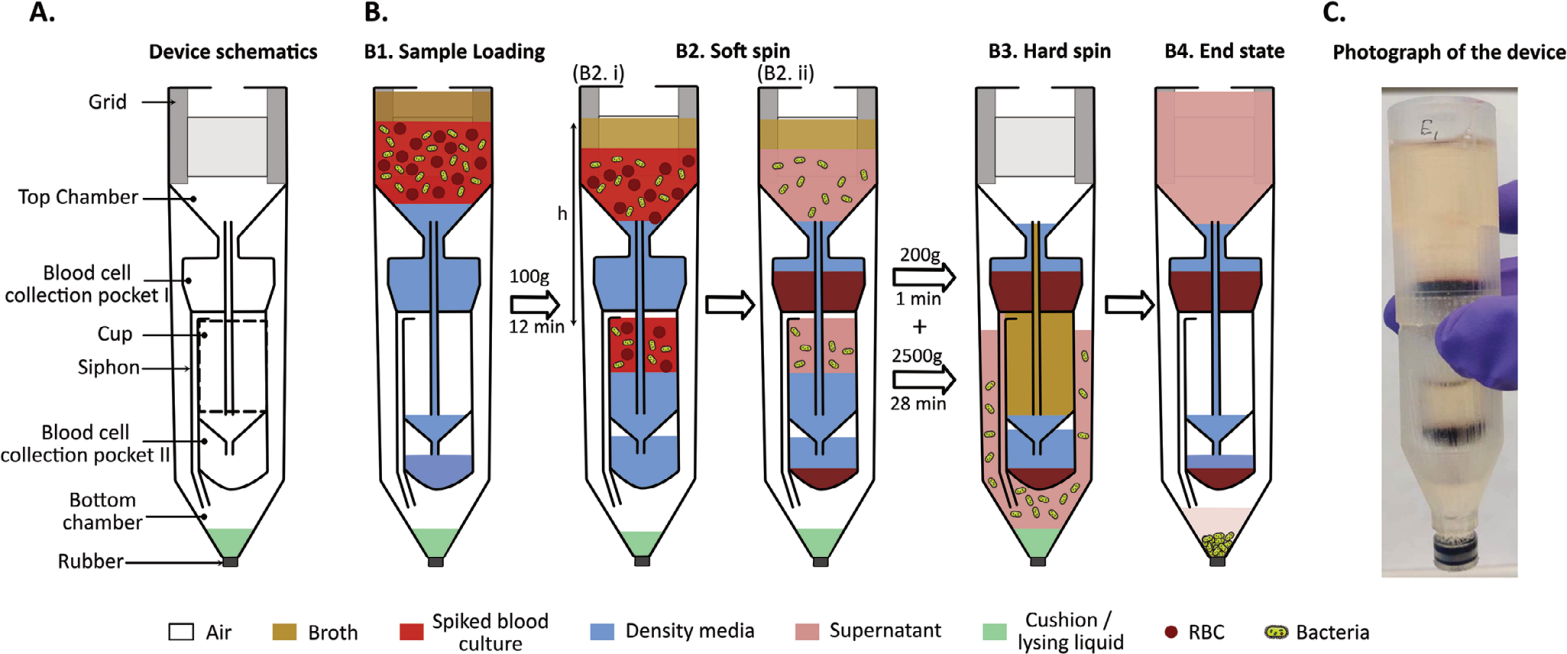
Rapid and automated isolation of bacteria from blood with a centrifuge‐based device. A) Cross‐sectional schematic of the device. The design of the device with different design features is highlighted, and the bottom chamber is pre‐filled with cushion/lysing liquid. B) Operation of the device. B1) Sample loading. The top chamber was first filled with density media, and the spiked blood mixed with culture media was placed on top of it, followed by the addition of broth media. B2) Soft spin. The device is centrifuged at 100g to settle the blood cells in the collection pocket while preventing liquid transport to the bottom chamber. B3) Hard spin. The device is centrifuged at high g to move the supernatant to the bottom chamber and sediment the remaining blood cells and bacteria. B4) End state. The centrifuge is stopped to transfer the liquid back from the bottom to the top chamber, leaving a small volume of liquid in the bottom chamber. C) Photograph of a device after bacterial separation.

We developed a centrifuge‐based device for the automated sample preparation of blood samples from septic patients, designed to facilitate the downstream microbiological assays of 1) bacterial subculturing, 2) MALDI‐TOF‐based bacterial identification, or 3) microtrap‐based bacterial detection. The device has the form factor of a standard 50 mL centrifuge tube and consists of a top and a bottom chamber connected by a siphon (**Figure** **1**A). The top chamber contains a grid, a cup‐like structure, and two blood cell collection pockets. The bottom chamber features a rubber stop in its base. Bacterial separation and concentration are achieved in a single centrifugation protocol by concatenating velocity‐based sedimentation of blood cells, automated transport of supernatant with bacteria, and sedimentation of bacteria in supernatant.

### 2.2 Device Preparation

The devices were 3D printed. The bottom chamber was pre‐filled with 0.4 mL of either a lysing solution or a cushion liquid, depending on the intended downstream application. The top chamber was pre‐filled with 5 mL of a density medium composed of a mixture of Lymphoprep and blood culture medium (BCM) in a ratio of 3:5. Air trapped in the cup and bottom chamber prevents the density medium from flowing down to the bottom chamber. 7.5 mL blood mixture, consisting of 3 mL spiked blood and 4.5 mL BCM, was layered on top of the density medium. 5 mL broth medium was layered on top of the sample.

### 2.3 Centrifuge Operation

For a constant centrifugal acceleration, $g^{*}$, and fluid density, ρ, the pressure in the trapped air in the bottom, $P_{b}$, relates to the hydrostatic height difference in the device, h, as

(1) $$P_{b}=P_{atm}+\rho \cdot g^{*}\cdot h$$

Our centrifugation protocol proceeds with a first soft spin step followed by a second hard spin step, as shown in Figure 1. The soft spin at 100 g for 12 min moves fluid into the cup and compresses the trapped air, such that the fluid fills the cup but does not enter the bottom chamber. Blood cells exhibit terminal sedimentation velocities 20–30 times higher than bacterial cells,^[^ ^26^ ^]^ resulting in the blood cells sedimenting into the blood collection pockets while the bacteria remain in the supernatant.

During the hard spin, the device is first accelerated to 200g for 1 min and then 2500g for 28 min. Increasing the spin speed above a critical centrifugal acceleration, $g_{crit}$, initiates fluid transfer from the cup to the bottom chamber. $g_{crit}$ can be designed for the range 100g < $g_{crit}$ < 200g with an appropriate device geometry (cup volume, $V_{c}$, and bottom chamber volume, $V_{b}$) resulting in a hydrostatic height, *h*, for which

(2) $$\rho \cdot g_{crit}\cdot h=P_{atm}\cdot \frac{V_{c}}{V_{b}}$$

The centrifugation at 200g gently transfers supernatant to the bottom chamber without stirring the blood cell sediment in the collection pockets. The broth medium fills the cup, ensuring that all bacteria‐containing liquid is transferred to the bottom chamber. During liquid transfer to the bottom chamber, the trapped air is further compressed until a new equilibrium is obtained between the hydrostatic pressure and the pressure in the compressed air volume. We verified that the liquid volume transported to the bottom chamber was consistently in the range between 8.6 and 10 mL (see Supporting Information for details). Increasing the centrifugation to 2500g facilitates the efficient sedimentation of bacterial cells in the bottom chamber. When the centrifuge stops, air decompression in the bottom chamber drives all the liquid above the siphon tube back to the top chamber, leaving the sedimented bacteria in a strongly reduced liquid volume in the bottom chamber, separated from the cup by air in the siphon. The device was then vortexed to resuspend the bacteria in the bottom chamber, after which the liquid was sampled from the bottom chamber using a syringe that pierced through the rubber stopper.

### 2.4 Concatenating Bacterial Isolation with Subculturing

**Figure 2:**
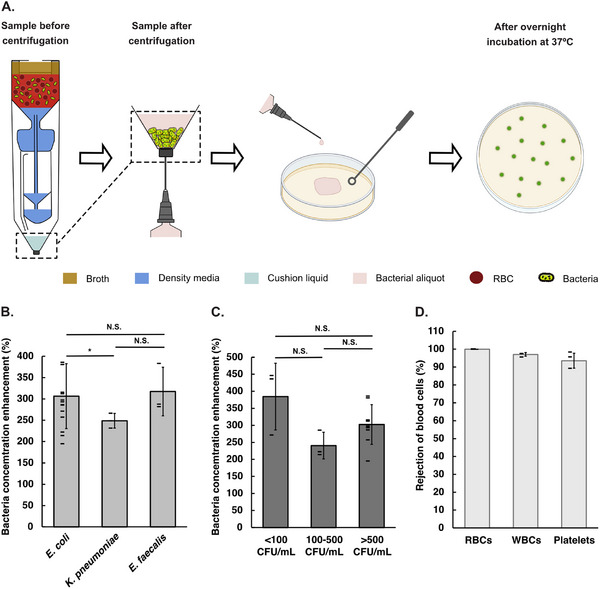
Centrifugal isolation concatenated with subculture. A) Process flow. B) Bacterial concentration enhancement, being the concentration of bacteria in the final aliquot relative to that in the spiked blood sample. C) Bacterial concentration enhancement, being the concentration of bacteria in the final aliquot relative to that in the spiked blood sample, classified into three ranges of bacteria concentration for *E. coli*. D) Rejection of blood cells, being the number of blood cells in the top and cup after centrifugation, relative to the initial number of blood cells in the blood sample. Error bars are sd; p‐Value (tails 2, type 3) indicates significance: N.S. is not significant, * is *p* < 0.05.

Bacterial subculturing following isolation is a key step for obtaining pure colonies, enabling accurate identification and AST. To assess the potential of our centrifugal isolation method, we evaluated its performance in combination with subsequent bacterial subculturing (**Figure** **2**). The bottom chamber was prefilled with 0.4 mL cushion fluid. The above‐described centrifugation protocol resulted in approximately 0.7 mL of up‐concentrated bacteria in the bottom chamber. The entire device was vortexed to resuspend the bacteria in the bottom chamber. The sample was extracted with a syringe, plated on agar plates, and left for overnight culture.

All experiments resulted in positive detection. The bacterial concentration enhancement, i.e., the concentration of bacteria in final aliquot relative to bacterial concentration in the spiked blood sample, were 302±58% (n = 9), 240±39% (n = 3), and 384±98% (n = 3) for concentrations of 500, 100, and 10 CFU/mL, respectively, of *E. coli*. The bacterial concentration enhancement for *K. pneumoniae* and *E. faecalis* at concentration of 500 CFU/mL were 249±17% (n = 3) and 317±57% (n = 3), respectively. The difference in bacterial concentration enhancement is significant only between *E. coli* and *K. pneumoniae*; other differences are not significant. The rejection percentages of blood cells are 99.97±0.01% (n = 4) for red blood cells, 97±1% (n = 4) for white blood cells, and 94±4% (n = 4) for platelets.

### 2.5 Concatenating Bacterial Isolation with MALDI‐TOF‐Based Identification

**Figure 3:**
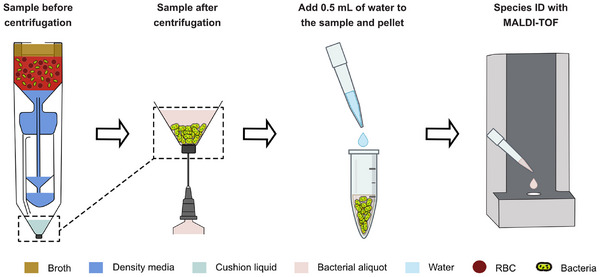
Centrifugal isolation concatenated with MALDI‐TOF. A) Centrifugal process flow for sample preparation for MALDI‐TOF. B) MALDI‐TOF scores for three bacterial species (*E. coli*, *K. pneumoniae* and *P. piersonii*). Scores between 2 and 3 indicate high‐confidence identification; scores between 1.7 and 1.99 indicate low‐confidence identification; and scores below 1.7 represent failure to identify any species.

We evaluated the potential of our new isolation approach for MALDI‐TOF sample preparation (**Figure** **3**A). The bottom chamber was prefilled with 0.4 mL of cushion fluid. A volume of 3 mL of blood was mixed with 4.5 mL of overnight bacterial culture, with each bacterial species (*E. coli*, *K. pneumoniae*, and *P. piersonii*) tested separately. The resulting sample for each test had an initial bacterial concentration of approximately $5\times 10^{8}$ CFU/mL. The 0.7 mL sample retrieved after centrifugation was mixed with 0.5 mL of deionized (DI) water and incubated at 37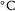 for 10 min. The mixture was centrifuged at 4000g for 5 min, and the resulting pellet was resuspended in 150 μL of solution. A 1 μL aliquot was transferred onto a MALDI target plate and air‐dried at room temperature, followed by the application of the matrix solution and another air‐dry, followed by analysis using a MALDI‐TOF MS system. The identification score, or log score, was calculated by the MALDI‐TOF MS machine based on the match between the sample's spectrum and reference spectra. *E. coli* and *K. pneumoniae* were identified with high confidence, and the *P. piersonii* with low confidence (n = 4), as shown in Figure 3b.

### 2.6 Concatenating Bacterial Isolation with Microtrap‐Based Bacteria Detection

**Figure 4:**
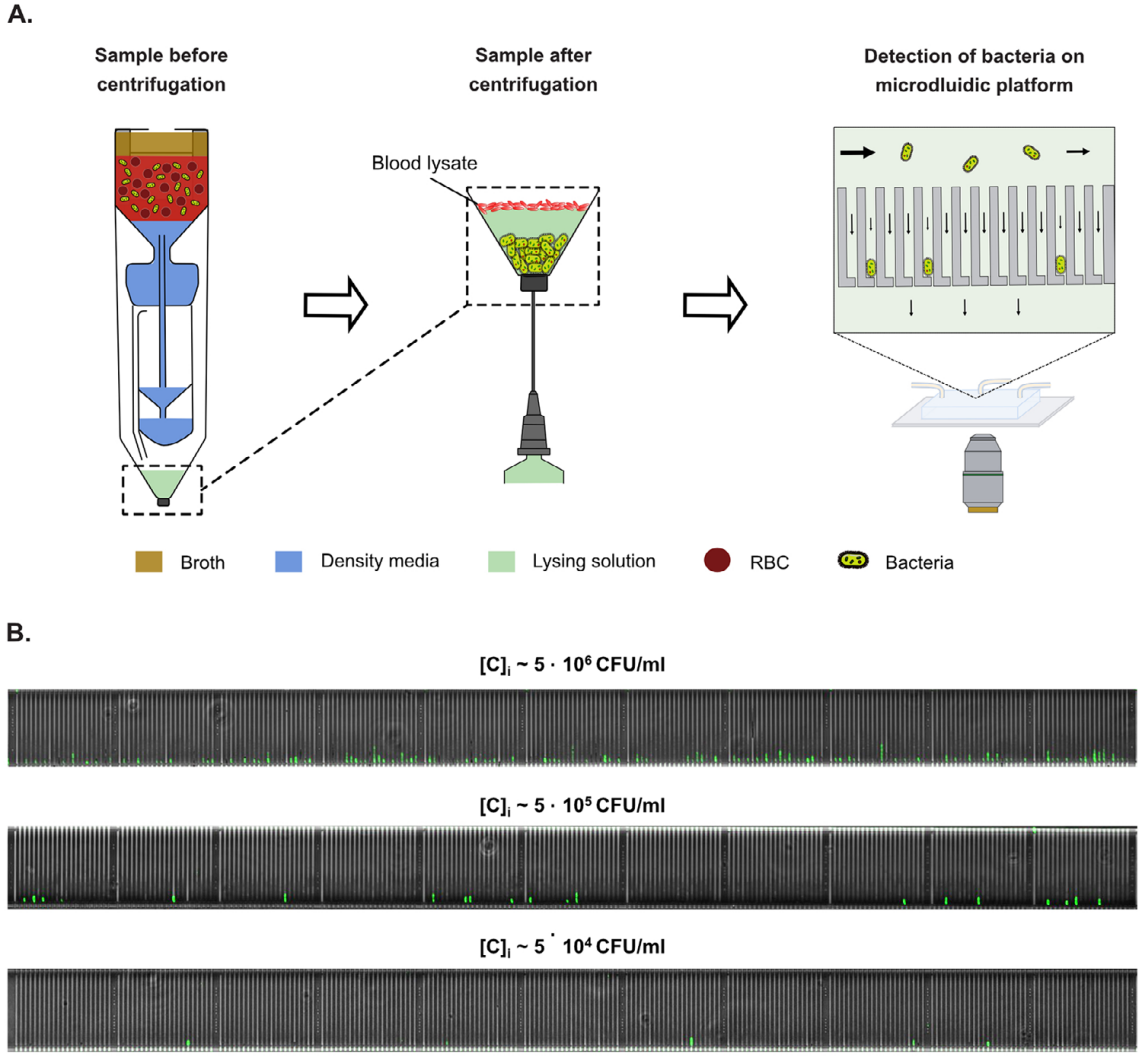
Centrifugal isolation concatenated with microtrap‐based detection. A) Centrifugal process flow for sample preparation for microtrap‐based detection. The bottom chamber of the device was prefilled with lysing solution. After centrifugation, the final liquid was sampled from the device and loaded onto a microfluidic chip. The solid arrows represent flow velocity. B) Fluorescence microscopy images of the microtraps with GFP‐labeled *E. coli* trapped in the microtraps with three different concentrations of spiked bacteria: $5\times 10^{6}$, $5\times 10^{5}$, and $5\times 10^{4}$ CFU/ml.

We evaluated the potential of our new isolation approach for microtrap‐based detection, we pre‐filled the bottom chamber with 0.4 mL of lysing solution dissolved in Percoll in a 5:3 volumetric ratio (**Figure** **4**A). Blood samples spiked with three different concentrations ($5\times 10^{6}$, $5\times 10^{5}$, and $5\times 10^{4}$ CFU/ml) of Green Fluorescent Protein (GFP)‐labeled *E. coli* were used. During hard spin centrifugation, supernatant liquid in the bottom chamber remained on top of the denser lysing solution. The centrifugation process sedimented the bacterial cells and remaining blood cells into the lysing solution, resulting in the selective lysis of the blood cells. Notably, Percoll, a density gradient medium, forms a density gradient during centrifugation, while the lysing solution remains uniformly distributed due to proper mixing at the outset. The 0.7 mL sample retrieved after centrifugation was resuspended, incubated for 10 min, and loaded into a previously developed microtrap platform.^[^ ^24^ ^]^ For all experiments, *E. coli* cells were successfully detected using fluorescent microscopy.

## 3 Discussion

We developed a centrifuge‐based device for the automated high‐throughput isolation and concentration of bacteria from blood for rapid sepsis diagnosis. Combining this sample preparation with downstream assays allows for faster, less labor‐intensive, and low‐contamination‐risk bacterial detection and identification compared to state‐of‐the‐art workflows.

The design of the device, including the cup, siphon and the trapped air, which acts as a pneumatic valve, allows controlling the transport and metering of liquid volumes by varying the centrifugation parameters. Previously, air‐compression‐based valves have been used in lab‐on‐disk systems;^[^ ^39^ ^]^ however, these systems were limited in throughput, required non‐standard laboratory equipment, and needed active valves. Our device addresses these limitations by handling 3 ml of blood and potentially 17.5 mL of blood culture; and operating on standard centrifuges without additional valves. During device design, we needed to solve specific challenges arising from large device size, specifically vacuum boiling, blood cell resuspension during fluid transport, and large dead volumes. These challenges were addressed through the careful selection of design parameters for the cup, siphon, blood cell collection pockets, and grid, as follows:

1. Design and position of the cup and siphon: The cup constitutes an internal fluid volume that allows partial compression of the trapped air without liquid transfer to the bottom chamber during the soft spin. The siphon entry is placed at the top of the cup, such that the entire air volume in the cup, $V_{c}$, can be used for compression. The cup is positioned such that its entire lumen remains below the top liquid surface at any time during operation, thus avoiding negative hydrostatic pressure leading to liquid boiling (details in Supporting Information). Increasing the cup volume allows increasing the relative centrifugal force (RCF) during the soft spin without transferring liquid to the bottom chamber, as described in Equation (2). A large cup volume, however, forms a dead volume for the liquid transferred from the top chamber to the bottom chamber, risking the trapping of bacteria in the cup during their intended transfer to the bottom chamber. To mitigate the latter, a layer of broth is added above the spiked blood mixture such that during hard spin, the broth fills the entire lumen of the cup, thereby driving the bacteria‐rich supernatant into the bottom chamber.
2. Blood cell collection pocket design: The connections to the blood cell collection pockets were kept narrow to reduce the swirling of blood cells during centrifugal acceleration or deceleration, hence avoiding unwanted resuspension and blood cell transfer to the bottom chamber.
3. Grid design: To prevent the broth from mixing with the supernatant during low‐speed to high‐speed centrifugation acceleration, a rectangular grid‐like structure was introduced at the top of the top chamber.^[^ ^29^ ^]^ Additionally, the cross‐sectional area of the lower part of the top chamber was reduced. These modifications minimize the effects of Coriolis and Euler forces, which could otherwise mix the sample and reduce the efficiency of velocity‐based sedimentation.
4. Preloaded liquids for separation: To isolate bacteria from blood, the spiked blood mixture was layered over density media, facilitating separation based on velocity differentiation. The falling distance through the density media enhances the separation of bacteria from blood cells. Further, a cushion liquid in the bottom chamber minimizes bacterial loss due to excessive centrifugation.
5. Liquid transfer analysis: In all the above experiments, the top chamber was filled with 17.5 mL of liquid. During the hard spin, between 8.6 and 10 mL of liquid was transferred to the bottom chamber, while 8–9 mL remained in the top chamber and cup (Figure S2, Supporting Information). Of the liquid remaining in the top chamber, approximately 4 mL was retained in the blood cell collection pockets, approximately 4 mL in the cup, and the rest constituted the dead volume above the blood cell collection pockets. The liquid retained in the blood cell collection pockets contained mostly blood cells, whereas the cup‐like structure was filled with broth media, which should contain few bacterial cells as it remains at the top of the blood mixture.

The state‐of‐the‐art methods for isolating and concentrating bacteria from blood have significant limitations, including low up‐concentration factors^[^ ^26^, ^27^, ^29^, ^32^, ^33^, ^34^, ^35^ ^]^ (the ratio of bacterial concentration in the final aliquot to the initial sample) or limited throughput^[^ ^29^, ^31^, ^32^, ^33^, ^34^, ^35^, ^36^ ^]^ (Table S1, Supporting Information). These challenges hinder their clinical use, as diagnosing sepsis or BSIs requires addressing extremely low bacterial concentrations in clinical samples. Our approach addresses these challenges with an up‐concentration factor above three and a per‐device sample throughput of 3 ml blood in 40 min, i.e., 75 μLmin ^−1^, outperforming most existing methods. In contrast to many methods that work well only at higher bacterial concentrations,^[^ ^32^, ^33^, ^35^, ^36^ ^]^ our approach works reliably even at 10 CFU/mL. While most methods focus only on bacterial isolation, ours performs full sample preparation, including bacterial concentration and selective cell lysis. Additionally, our device operation is scalable, with up to 16 devices operating simultaneously in centrifuges equipped with 16 holders, such as the Thermo Scientific Sorvall ST 16 Centrifuge, thus supporting high‐throughput processing. These benefits make our approach a practical and efficient solution for clinical diagnostics.

Subculturing is a method for obtaining pure solid bacterial colonies, which are required for MALDI‐TOF and genotypic‐based detection. Our method prepares blood samples for subculturing in less than 1 h, even at bacterial concentrations as low as 10 CFU/mL, reducing the procedure time by hours to days compared to the current praxis requiring a priori positive blood cultures.

We successfully demonstrated the suitability of our device for sample preparation for MALDI‐TOF analysis. The score for *P. piersonii* was low, likely due to the low concentration of bacterial cells in the overnight culture. This limitation can be mitigated by using a higher bacterial concentration. In most clinical settings, standard MALDI‐TOF sample preparation requires subculturing of positive blood cultures—a process that takes 6–12 h and can delay appropriate antibiotic therapy by up to a day.

In contrast, our device produces purified bacterial aliquots in less than 1 h, directly suited for MALDI‐TOF‐based identification, enabling accurate determination of the causative bacterial species with high score values. In contrast to previous method, which required at least 5.5 h of incubation on solid media prior to MALDI‐TOF MS analysis,^[^ ^16^ ^]^ our device produces purified bacterial aliquots in less than 1 hour that are directly suited for MALDI‐TOF identification and enable accurate determination of the causative bacterial species with high score values. Furthermore, a detailed comparison between state‐of‐the‐art sample preparation kits, such as Sepsityper^[^ ^18^, ^19^ ^]^ and the Accelerate Arc System,^[^ ^19^, ^20^ ^]^ and our device is presented in **Table** **1**. Compared to Sepsityper,^[^ ^18^, ^19^ ^]^ our device offers the advantages of reduced hands‐on time, and minimal user dependency. While the Accelerate Arc System^[^ ^19^, ^20^ ^]^ requires a specialized system, our device only needs a standard centrifuge to operate. Another limitation of both methods is the mandatory use of a lysing agent during sample preparation for MALDI‐TOF. While effective for identification, these agents can inhibit bacterial growth—particularly of Gram‐positive species—making them less suitable for rapid antimicrobial susceptibility testing (AST).^[^ ^21^, ^22^ ^]^ In contrast, our device offers the flexibility to perform sample preparation with or without a lysing agent, making it more adaptable and better suited for workflows requiring rapid AST.

**Table 1:** Comparison of our device with state‐of‐the‐art sample preparation kits for MALDI‐TOF MS. The comparison includes cost, hands‐on time, compatibility with AST, and the need for specialized equipment.

Methods	Hands‐on‐time (min)	Compatible with rapid AST	Requires specialized equipment
This Study	≈5	Yes	No
Sepsityper	≈10	No	No
Accelerate Arc System	<5	No	Yes

We also successfully demonstrated the suitability of our device for preparing samples for bacterial detection on microtrap platforms. Microfluidics‐based bacterial culture is a promising technology with the potential to significantly advance diagnostic methods, particularly in its ability to perform rapid phenotypic testing by analyzing the growth of single bacterial cells, unlike traditional clinical practices that rely on macroscopic growth.^[^ ^24^ ^]^ Additionally, bacteria can be identified on such platforms using genotypic methods such as FISH.^[^ ^40^ ^]^ However, microfluidic devices typically require pure bacterial cultures, as interfering cells readily clog the microtraps—especially those with dimensions of just a few microns—thereby compromising the assay. Current methods and devices for sample preparation from whole blood for microfluidic‐based detection are multiple‐step, labor‐intensive and require trained personnel.^[^ ^27^, ^28^ ^]^ In contrast, our device simplifies bacterial isolation by automating multiple centrifugation steps and blood cell lysis in a single centrifugation protocol. Moreover, initial bacterial concentrations as low as $5\times 10^{4}$ CFU/ml yielded positive detection, which is significantly lower than the levels typically found in positive blood cultures.

Further, with a view toward translation, we speculate that this device could be readily adapted for mass production, for example, using low‐cost injection molding‐based techniques. We further speculate that an end‐user version of the device could be pre‐loaded with cushion liquid, density media, and broth, requiring the end user to simply add blood or blood culture before use. Also, validation with a broader panel of clinically relevant bacterial strains, including opportunistic pathogens such as *Stenotrophomonas maltophilia*, will be important to establish broad applicability.

## 4 Conclusion

We have developed an automated, centrifuge‐only device that redefines sample preparation for sepsis diagnostics. Operating with standard clinical centrifuges and requiring no manual intervention or active components, this platform streamlines a traditionally multi‐step, labor‐intensive workflow into a single centrifugation protocol completed within 40 min. Compared to current assays, ours significantly reduces the need for trained personnel. Our system achieves more than threefold bacterial enrichment while removing >99.9% of blood cells, delivering assay‐ready samples even from ultra‐low concentrations of 10 CFU/mL. It seamlessly feeds into critical downstream diagnostics—including subculturing, MALDI‐TOF identification, and microfluidic single‐cell analysis—with significantly reduced turnaround time and contamination risk. By enabling culture‐free, same‐shift pathogen identification, this technology sets a new standard for rapid, scalable, and clinically compatible blood processing. Its adoption could fundamentally accelerate infection management, improving patient outcomes while alleviating pressure on healthcare systems worldwide.

## 5 Experimental Section

### Device Fabrication

The device was designed using SolidWorks CAD software and fabricated in three distinct parts: top, bottom, and cap, as detailed in Figure S3 (Supporting Information). Fabrication was performed using a Form 3+ 3D printer (Formlabs, USA) with Clear V4 resin. Post‐fabrication, the components were cleaned using a Form Wash (Formlabs, USA) with isopropanol (IPA) to remove residual resin from the surfaces. The internal structures of the device were manually cleaned using a needle and IPA to ensure thorough removal of excess resin. Subsequently, the parts were cured in a Form Cure (Formlabs, USA) using ultraviolet (UV) light for 30 min at 60 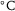. To address challenges during 3D printing, a hole was incorporated into the blood cell collection pocket II of the top part to prevent cupping. This hole was sealed post‐fabrication using Clear V4 resin to prevent leakage during operation.

### Device Assembly

The top and bottom 3D‐printed components were adhered using Clear V4 resin, which was solidified through curing with UV light in the Form Cure for 10 min. A polyisoprene rubber plug was inserted into the cap and glued using ClearSeal Glass Clear adhesive (Casco, Switzerland), then allowed to dry overnight. Subsequently, the cap was attached to the bottom component using Clear V4 resin. Prior to this step, the bottom chamber was pre‐filled with a cushion or lysing liquid according to the use case.

### Medium Preparation

The density medium used in this study was a mixture of Lymphoprep (STEMCELL Technologies, Canada), a medium with a density of 1.077 gmL^−1^, and blood culture medium (BCM) (BD BACTEC Plus Aerobic medium, BD, USA) in a 3:5 ratio. The broth was prepared by dissolving Luria low salt powder (L3397, Sigma‐Aldrich, USA) in deionized water (DIW) at a concentration of 25 gL^−1^, followed by autoclaving to ensure sterility. The cushion liquid employed was a combination of Percoll (Sigma‐Aldrich, USA), a density medium with a density range of 1.125–1.135 gmL^−1^, and BCM mixed in a ratio of 3:5. The lysing solution used in the study was prepared by mixing Percoll with a lysing liquid in a 3:5 ratio. The lysing liquid itself consisted of 2% (wv^−1^) sodium cholate hydrate (Sigma‐Aldrich, USA) and 1% (wv^−1^) saponin (Sigma–Aldrich, USA), both dissolved in BCM.

### Bacterial Strains

Four different bacterial strains were used in this study. The *E. coli* strain carried a plasmid expressing mVenusNB fluorescence proteins. The other bacterial strains, *K. pneumoniae*, *E. faecalis*, and *Pantoea piersonii*, were clinical strains randomly collected from a clinical microbiology laboratory in Sweden. For long‐term storage, the bacteria were maintained at ‐80 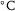 in standard glycerol solution. Prior to use, the strains were incubated overnight at 37 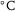 in BD BACTEC Plus Aerobic medium (BD, USA), referred to as Blood Culture Medium (BCM). After incubation, the bacterial cultures were diluted in BCM to approximately $10^{4}$, $10^{3}$, and $10^{2}$ CFU/mL, which were subsequently used to spike blood samples at varying concentrations for the experiments. Further, the concentration of the spiking solution was determined by plate counting of bacterial solution of $10^{3}$ CFU/ml.

### Spiked Blood Preparation

In the experiments, healthy donor blood was obtained from the blood bank (Blodcentralen, Stockholm, Sweden). The blood samples were used within two days of collection and stored at 4 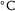 prior to use. The blood was diluted with BCM in a 2:3 ratio and spiked with bacteria. During spiking, the volume of the liquid added was always less than 4% of the total volume of the blood culture.

### Bacteria Counting

Bacterial counting was conducted by plating the bacterial solution on agar plates, followed by overnight incubation at 37 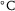. The agar plates were prepared by dissolving LB broth with agar (Miller) (Sigma‐Aldrich, USA) in DIW at a concentration of 40 g/L. The mixture was autoclaved, poured into Petri dishes, and cooled. For all subculture experiments, 100 μL of the final aliquot was plated in triplicates. However, the entire final aliquot was plated in the 10 CFU/mL experiments to ensure accurate quantification.

### Blood Cell Quantifiation

Blood cell counts in whole blood and final aliquots were measured using a hematology analyzer (Swelab Alfa Plus, Boule Diagnostics, Sweden).

### Process for MALDI‐TOF

A 1 μL aliquot of the final sample was applied to the MALDI‐TOF plate and air‐dried at room temperature. Subsequently, the matrix solution (α‐cyano‐4‐hydroxycinnamic acid) was applied, and the sample was analyzed using the MALDI‐TOF MS system after drying the matrix solution. The MALDI‐TOF machine used in the study was MALDI Biotyper Sirius One System (Bruker Daltonics, Bremen, Germany).

### Microfluidic Platform Fabrication

The design and the fabrication of the microfluidic chip were previously reported.^[^ ^24^, ^41^ ^]^ The silicon mold used for the chip was manufactured by ConScience AB, Sweden, and was silanized for 30 min before replication with polydimethylsiloxane (PDMS) (Sylgard 184, DOW, USA). A mixture of PDMS and curing agent in a 10:1 w/w ratio was poured onto the mold and cured at 80 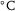 overnight. Openings for the PDMS ports (2.0, 2.1, 2.2, 5.1, and 5.2; see chip design in Ref. [24, 41]) were created using a 0.5 mm puncher. The fabricated PDMS stamps were cleaned with isopropanol (IPA) before being bonded to a glass coverslip (No. 1.5, Menzel‐Gläser, Germany) using plasma treatment. The bonded assembly was subsequently heat‐cured for an h at 80 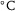.

### Flow Control

The microfluidic device was set up on a microscope and connected to reservoirs using tubing (TYGON, Saint‐Gobain, North America). The flow controller used for pressurizing the reservoirs was the FlowEZ (Fluigent, France). For priming, the reservoirs connected to ports 2.1, 2.2, 5.1, and 5.2 were initially pressurized at 500 mbar with water containing 0.085 g/L of Pluronic F108 (Sigma‐Aldrich, USA), after which the pressure was reduced to 0 mbar. Subsequently, the reservoir connected to inlet port 2.0 was pressurized at 500 mbar for priming and then increased to 1000 mbar for sample loading.

### Optical Setup

Images of the microtraps were captured at 100x magnification using a Nikon Eclipse Ti‐U inverted microscope. Fluorescence images were also taken at 100x magnification using a CFI Plan Fluor DLL 100x (1.30 NA, oil) objective. The fluorescence images were acquired with a filter cube comprising a FF497‐Di01 dichroic mirror (Semrock, USA), a FF01‐469/35 excitation filter (Semrock, USA), and a FF01‐525/39 emission filter (Semrock, USA).^[^ ^42^ ^]^

### Statistical Analysis

The data analysis was conducted using Microsoft Excel v16.0. It was assumed that the data was normally distributed and that the variances were unequal across samples. To identify significant differences between group averages, a two‐tailed t‐test of type 3 was used.

### Ethical Statement

We used anonymized blood obtained from healthy donors through a certified blood bank, solely for technical development purposes, and therefore, no specific ethical approval was required under Swedish regulations.

## Conflict of Interest

M.O and W.W. have commercial interests in the diagnostic field. M.O, W.W and M.H.M have filed a patent application based on the device presented in this paper.

## Author Contributions

M.O and W.W conceptualized the study. W.W and M.O designed the device. M.O designed the experimental protocol. M.O, M.H.M, B.B, and B.B.O performed the experiments. M.O conducted the data analysis. M.O and M.H.M prepared the figures. The manuscript was written by M.O, with critical review and editing by W.W, V.Ö, M.H.M, B.B, and B.B.O. Project supervision and funding for the study were provided by W.W. All authors read and approved the final manuscript.

## Supporting information

Supporting Information

Supplemental Movie 1

Supplemental Movie 2

Supporting Information

## Data Availability

The data that support the findings of this study are available in the supplementary material of this article.

## References

[1] K. E. Rudd , S. C. Johnson , K. M. Agesa , K. A. Shackelford , D. Tsoi , D. R. Kievlan , D. V. Colombara , K. S. Ikuta , N. Kissoon , S. Finfer , C. Fleischmann‐Struzek , F. R. Machado , K. K. Reinhart , K. Rowan , C. W. Seymour , R. S. Watson , T. E. West , F. Marinho , S. I. Hay , R. Lozano , A. D. Lopez , D. C. Angus , C. J. L. Murray , M. Naghavi , Lancet 2020, 395, 200. 31954465 10.1016/S0140-6736(19)32989-7 PMC6970225

[2] R. S. Hotchkiss , L. L. Moldawer , S. M. Opal , K. Reinhart , I. R. Turnbull , J.‐L. Vincent , Nat. Rev. Dis. Primers 2016, 2, 1. 10.1038/nrdp.2016.45 PMC5538252 28117397

[3] K. Thompson , B. Venkatesh , S. Finfer , Intern. Med. J. 2019, 49, 160. 30754087 10.1111/imj.14199

[4] L. E. Huerta , T. W. Rice , J. Appl. labor. Med. 2019, 3, 654. 10.1373/jalm.2018.026245 31639733

[5] D. W. Bates , K. Sands , E. Miller , P. N. Lanken , P. L. Hibberd , P. S. Graman , J. S. Schwartz , K. Kahn , D. R. Snydman , J. Parsonnet , R. Moore , E. Black , B. L. Johnson , A. Jha , R. Platt , J. Infect. Dis. 1997, 176, 1538. 9395366 10.1086/514153

[6] A. Kumar , D. Roberts , K. E. Wood , B. Light , J. E. Parrillo , S. Sharma , R. Suppes , D. Feinstein , S. Zanotti , L. Taiberg , D. Gurka , A. Kumar , M. Cheang , Crit. Care Med. 2006, 34, 1589. 16625125 10.1097/01.CCM.0000217961.75225.E9

[7] H. Arefian , S. Heublein , A. Scherag , F. M. Brunkhorst , M. Z. Younis , O. Moerer , D. Fischer , M. Hartmann , J. Infect. 2017, 74, 107. 27884733 10.1016/j.jinf.2016.11.006

[8] C. Rhee , T. M. Jones , Y. Hamad , A. Pande , J. Varon , C. O'Brien , D. J. Anderson , D. K. Warren , R. B. Dantes , L. Epstein , M. Klompas , JAMA Network Open 2019, 2, e187571. 30768188 10.1001/jamanetworkopen.2018.7571 PMC6484603

[9] C. Llor , L. Bjerrum , Ther. Adv. Drug. Saf. 2014, 5, 229. 25436105 10.1177/2042098614554919 PMC4232501

[10] N. G. Morgenthaler , M. Kostrzewa , Int. J. Microbiol. 2015, 2015, 827416. 26000017 10.1155/2015/827416 PMC4426779

[11] M. R. Jacobs , T. Mazzulli , K. C. Hazen , C. E. Good , A. M. Abdelhamed , P. Lo , B. Shum , K. P. Roman , D. C. Robinson , J. Clin. Microbiol. 2017, 55, 2413. 28539343 10.1128/JCM.00307-17 PMC5527419

[12] M. M. Lambregts , A. T. Bernards , M. T. van der Beek , L. G. Visser , M. G. de Boer , PLoS One 2019, 14, e0208819. 30601829 10.1371/journal.pone.0208819 PMC6314566

[13] Y. Kumar , M. Qunibi , T. Neal , C. Yoxall , Arch. Dis. Child. ‐ Fetal Neonatal Ed. 2001, 85, F182. 11668160 10.1136/fn.85.3.F182 PMC1721335

[14] E. M. Ransom , Z. Alipour , M. A. Wallace , C.‐A. D. Burnham , J. Clin. Microbiol. 2021, 59, 10. 10.1128/JCM.02459-20 PMC8106720 33239377

[15] N. Topić Popović , S. P. Kazazić , K. Bojanić , I. Strunjak‐Perović , R. Čož‐Rakovac , Mass Spectrom. Rev. 2023, 42, 1589. 34642960 10.1002/mas.21739

[16] O. Altun , S. Botero‐Kleiven , S. Carlsson , M. Ullberg , V. Özenci , J. Med. Microbiol. 2015, 64, 1346. 26361761 10.1099/jmm.0.000168

[17] K. C. Tjandra , N. Ram‐Mohan , R. Abe , M. M. Hashemi , J.‐H. Lee , S. M. Chin , M. A. Roshardt , J. C. Liao , P. K. Wong , S. Yang , Antibiotics 2022, 11, 511. 35453262 10.3390/antibiotics11040511 PMC9029869

[18] G. Perše , I. Samošćanec , Z. Bošnjak , A. Budimir , T. Kuliš , I. Mareković , Life 2022, 12, 1744. 36362899 10.3390/life12111744 PMC9693840

[19] J. H. Chen , P.‐L. Ho , G. S. Kwan , K. K. She , G. K. Siu , V. C. Cheng , K.‐Y. Yuen , W.‐C. Yam , J. Clin. Microbiol. 2013, 51, 1733. 23515548 10.1128/JCM.03259-12 PMC3716069

[20] B. Buchan , A. Cruz , M. Faron , D. Gerstbrein , B. Mesich , B. Sullivan , in Journal of Molecular Diagnostics, Elsevier Science INC STE 800, 230 Park Ave, New York, NY 10169 USA, vol. 24, 2022, pp. S51–S51.

[21] M. Hariu , Y. Watanabe , N. Oikawa , T. Manaka , M. Seki , Infect. Drug Resist. 2018, 1573. 30288067 10.2147/IDR.S169197 PMC6159798

[22] S. Cruz , D. Abreu , R. Gomes , I. Martins‐Oliveira , A. Silva‐Dias , B. Perez‐Viso , R. Cantón , C. Pina‐Vaz , European J. Clin. Microbiol. & Infectious Diseases 2024, 43, 605. 38112967 10.1007/s10096-023-04725-3 PMC10917851

[23] I. Gajic , J. Kabic , D. Kekic , M. Jovicevic , M. Milenkovic , D. Mitic Culafic , A. Trudic , L. Ranin , N. Opavski , Antibiotics 2022, 11, 427. 35453179 10.3390/antibiotics11040427 PMC9024665

[24] Ö. Baltekin , A. Boucharin , E. Tano , D. I. Andersson , J. Elf , Proc. Natl. Acad. Sci. USA 2017, 114, 9170. 28790187 10.1073/pnas.1708558114 PMC5576829

[25] M. Osaid , Y.‐S. Chen , C.‐H. Wang , A. Sinha , W.‐B. Lee , P. Gopinathan , H.‐B. Wu , G.‐B. Lee , Lab Chip 2021, 21, 2223. 33890605 10.1039/d1lc00216c

[26] W. G. Pitt , M. Alizadeh , G. A. Husseini , D. S. McClellan , C. M. Buchanan , C. G. Bledsoe , R. A. Robison , R. Blanco , B. L. Roeder , M. Melville , A. K. Hunter , Biotechnol. Prog. 2016, 32, 823. 27160415 10.1002/btpr.2299 PMC5297886

[27] M. H. Marino Miguélez , M. Osaid , E. Hallström , K. Kaya , J. Larsson , V. Kandavalli , C. Wählby , J. Elf , W. van der Wijngaart , npj Digit. Med. 2025, 8, 544. 40851034 10.1038/s41746-025-01948-w PMC12375708

[28] B. Forsyth , P. Torab , J.‐H. Lee , T. Malcom , T.‐H. Wang , J. C. Liao , S. Yang , E. Kvam , C. Puleo , P. K. Wong , Biosensors 2021, 11, 288. 34436090 10.3390/bios11080288 PMC8391654

[29] K. Zeng , M. Osaid , W. van der Wijngaart , Lab Chip 2023, 23, 4334. 37712252 10.1039/d3lc00594a

[30] M. H. Marino Miguélez , A. Huguenin‐Dumittan , M. Osaid , W. van der Wijngaart , Sci. Rep. 2025, 15, 24661. 40634447 10.1038/s41598-025-09024-9 PMC12241390

[31] T. H. Kim , J. Kang , H. Jang , H. Joo , G. Y. Lee , H. Kim , U. Cho , H. Bang , J. Jang , S. Han , D. Y. Kim , C. M. Lee , C. K. Kang , P. G. Choe , N. J. Kim , M. O , T. S. Kim , I. Kim , W. B. Park , S. Kwon , Nature 2024, 632, 893. 39048820 10.1038/s41586-024-07725-1

[32] P. Ohlsson , M. Evander , K. Petersson , L. Mellhammar , A. Lehmusvuori , U. Karhunen , M. Soikkeli , T. Seppa , E. Tuunainen , A. Spangar , P. V. Lode , K. R. Jalava , G. Otto , S. Scheding , T. Soukka , S. Wittfooth , T. Laurell , Anal. Chem. 2016, 88, 9403. 27264110 10.1021/acs.analchem.6b00323

[33] A. J. Mach , D. Di Carlo , Biotechnol. Bioeng. 2010, 107, 302. 20589838 10.1002/bit.22833

[34] S. Narayana Iyengar , T. Kumar , G. Mårtensson , A. Russom , Electrophoresis 2021, 42, 2538. 34510466 10.1002/elps.202100140

[35] S. Li , F. Ma , H. Bachman , C. E. Cameron , X. Zeng , T. J. Huang , J. Micromech. Microeng. 2016, 27, 015031. 28798539 10.1088/1361-6439/27/1/015031 PMC5546156

[36] S. Park , Y. Zhang , T.‐H. Wang , S. Yang , Lab Chip 2011, 11, 2893. 21776517 10.1039/c1lc20307j

[37] M. Kayin , B. Mert , S. Aydemir , V. Özenci , Eur. J. Clin. Microbiol. Infect. Dis. 2019, 38, 2133. 31494828 10.1007/s10096-019-03654-4 PMC6800852

[38] A. Croxatto , G. Prod'hom , C. Durussel , G. Greub , J. Vis. Exp. 2014, 92, 51985. 10.3791/51985 PMC4692429 25350577

[39] D. J. Kinahan , S. M. Kearney , N. A. Kilcawley , P. L. Early , M. T. Glynn , J. Ducree , PloS one 2016, 11, e0155545. 27167376 10.1371/journal.pone.0155545 PMC4864222

[40] V. Kandavalli , P. Karempudi , J. Larsson , J. Elf , Nat. Commun. 2022, 13, 6215. 36266330 10.1038/s41467-022-33659-1 PMC9584937

[41] D. Camsund , M. J. Lawson , J. Larsson , D. Jones , S. Zikrin , D. Fange , J. Elf , Nat. Methods 2020, 17, 86. 31740817 10.1038/s41592-019-0629-y

[42] E. Iseri , G. Jakobsson , S. Bertling , V. Özenci , O. Ekelund , W. van der Wijngaart , A. van Belkum , Eur. J. Clin. Microbiol. Infect. Dis. 2025, 1. 40063324 10.1007/s10096-025-05088-7

