# Supplementary material for: Plug‐and‐Play Centrifuge‐Only Device for Rapid Sepsis Diagnosis
Source: Adv Healthc Mater. 2025 Sep 2;14(32):e03651. doi: 10.1002/adhm.202503651 (PMC12716180; doi:10.1002/adhm.202503651)
Supplement: Supplementary file 1 — Supporting Information [file ADHM-14-0-s002.pdf]

ADVANCED HEALTHCARE MATERIALS

Supporting Information

for *Adv. Healthcare Mater.*, DOI 10.1002/adhm.202503651

Plug-and-Play Centrifuge-Only Device for Rapid Sepsis Diagnosis

*Mohammad Osaid, M. Henar Marino Miguélez, Berke Bayrak, Büsra Betül Özmen-Capin, Volkan Özenci and Wouter van der Wijngaart**

Supplementary Materials for

Plug-and-play centrifuge-only device for rapid sepsis diagnosis

Mohammad Osaid[1], M. Henar Marino Miguélez[1], Berke Bayrak[1],Büsra Betül Özmen-Capin[2], Volkan Özenci[2], Wouter van der Wijngaart[1,*]

[1]Micro and Nanosystems, KTH Royal Institute of Technology, Stockholm, Sweden.

[2]Department of Clinical Microbiology, Karolinska University Hospital, Huddinge, Sweden.

[*]Corresponding authors. Email: wouter@kth.se

This PDF file includes:

Supplementary Text

Figures S1 to S4

Tables S1

Captions for Movies

Captions for Data

Other Supplementary Materials for this manuscript:

Movies S1 to S2

Data S1

Setup to film the liquid motion in the device inside the moving centrifuge

An imaging setup was developed to monitor liquid motion within the device during centrifugation. A customized centrifuge holder was designed, 3D printed, and engineered to accommodate a camera, a light source, and the device, all of which fit inside a standard centrifuge bucket (Supplementary Figure S1). The holder incorporates an integrated wireless camera (Global Tsolar Lights Electrical, China), a light source (Ledlenser, Germany), and the fluidic device. During centrifugation, the wireless camera transmits real-time images of the process to an external computer, enabling continuous monitoring.

The device was filled with blood mixed with culture media and placed in the imaging setup. During centrifugation at 100g (soft spin), it was observed that the liquid level rose inside the cup-like structure, and the liquid level in the top chamber decreased, but no liquid was transferred to the bottom chamber (Supplementary Figure S1 A). However, during hard spin centrifugation at 2500g, the liquid was transferred to the bottom chamber, leaving only a residual volume in the cup-like structure. When the centrifuge was stopped, the liquid returned to the top chamber, leaving only a small amount of liquid in the bottom chamber.The video can be found in Supplementary files. The image of the imaging system is shown in (Supplementary Figure S1 B).

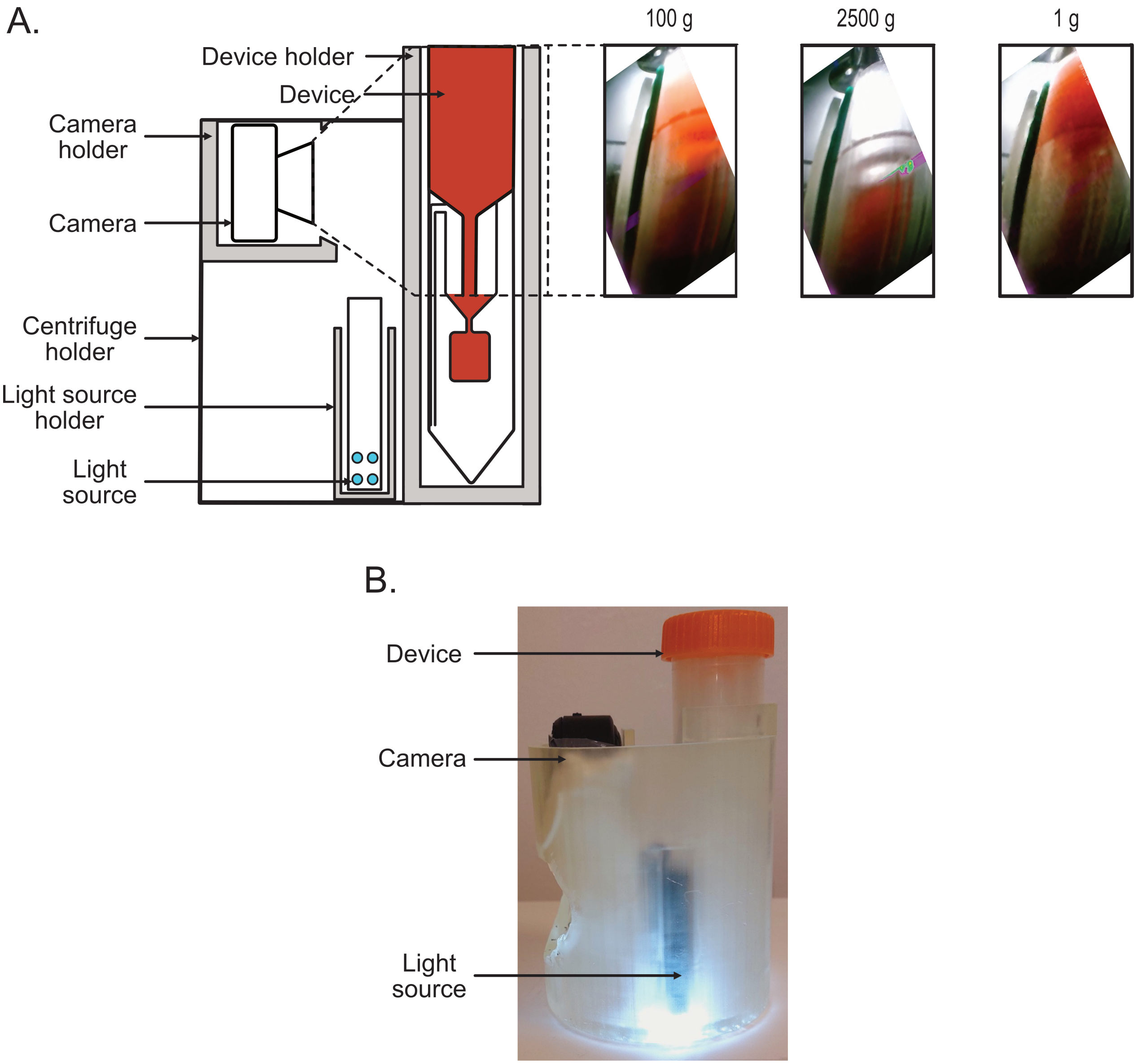

Figure S1: **Setup for filming inside a centrifuge tube: A.** Schematics of the holder used for filming the device inside the centrifuge, which includes compartments for the camera, the device, and the light source. On the right are the Images of liquid movement within the device during centrifugation taken from the camera. The figure includes snapshots of the device inside the centrifuge at 100g (soft spin), 2500g (hard spin), and 1g (centrifuge stopped). **B.** Photograph of the holder with the camera, the device, and the light source in place.

Calculation of Volume transferred to the bottom

The transfer of liquid to the bottom chamber was visualized using a camera placed inside the centrifuge tube during operation, as shown in Supplementary Figure S1. To precisely quantify the volume transferred, multiple small grooves or traps were introduced along the inner wall of the chamber. The volume below these traps, extending to the bottom of the tube, was measured manually. In other words, the bottom chamber of the tube was calibrated using these traps, as highlighted in Supplementary Figure S2.

During a hard spin, the liquid transferred to the bottom chamber filled the traps that became submerged. After filling the top chamber with 17.5 mL of liquid and centrifuging the device at 2500g, the first three traps from the bottom were filled, while the fourth trap at the top remained empty. The volumes corresponding to the regions below the third and fourth traps were 8.6 mL and 10 mL, respectively. Therefore, the volume of liquid transferred was determined to be between 8.6 mL and 10 mL.

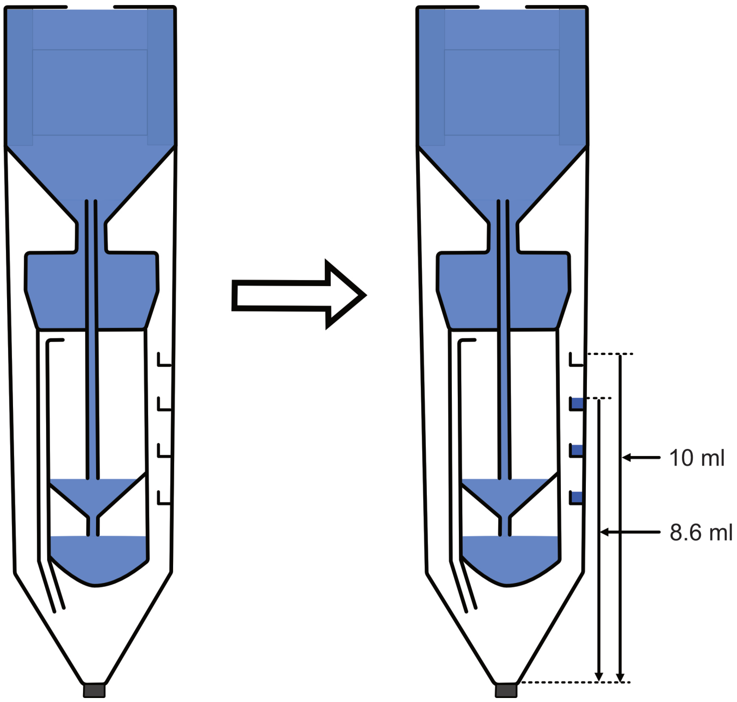

Figure S2: **Device design for quantifying the volume of liquid transferred to the bottom chamber.** Schematic of the device with microtraps in the bottom chamber. During hard spin, the liquid gets transferred to the bottom chamber and fills the microtraps. When centrifugation is stopped, the liquid returns to the top chamber, leaving the traps filled.

Device assembly

The device consists of three distinct 3D-printed components that are assembled together. The two larger parts, namely the Top and Bottom parts, as shown in Supplementary Figure S3 A, were bonded using clear v4 resin and subsequently cured in ultraviolet light in a Form Cure system (Formlabs, USA) for 10 min. The bottom cap incorporates a polyisoprene rubber plug sourced from 3 mL Megro™ SOFT-JECT™ disposable syringes (Henke-Sass, Wolf GmbH). This plug was attached to the 3D-printed cap using ClearSeal Glass Clear adhesive (Casco, Switzerland) and allowed to dry overnight. The cap was then affixed to the device using clear v4 resin and cured in ultraviolet light for an additional 10 min. Supplementary Figure S3 B illustrates the CAD model of the assembly of the device.

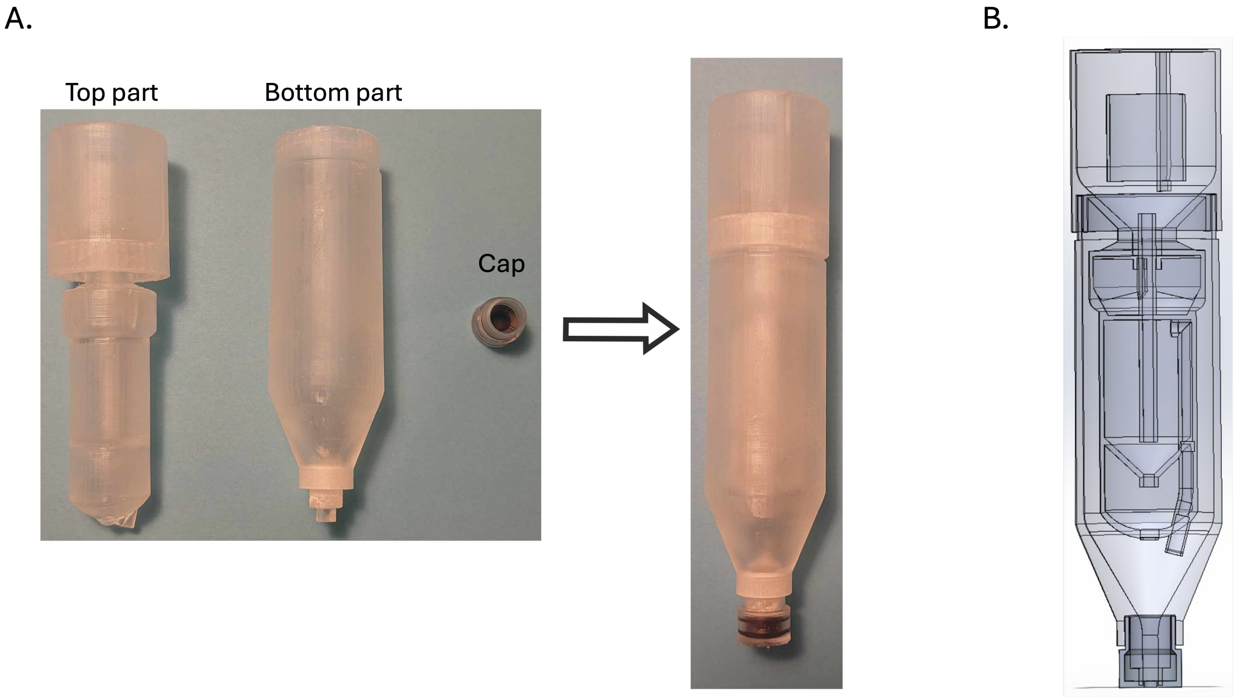

Figure S3: **Assembly of the 3D printed device. A.** Image showing the three separate 3D-printed components of the device, including the Top, Bottom, and cap, which are bonded using clear v4 resin and cured under ultraviolet light to form the final structure. **B.** CAD model illustrating the cross-section of the complete assembly of the device.

Vaccum creation in the centrifugal device

The device described in the article can be modified to create a vacuum by changing the position of the cup, i.e., by moving it upward in the top chamber, as shown in Supplementary Figure S4. The top chamber of the device is filled with liquid, and during a high-speed spin (centrifugation at 2500g), the liquid is transferred to the bottom chamber via the siphon, which consists of the cup and tube. Throughout the transfer process, the liquid surface in the top chamber remains at atmospheric pressure. When the liquid level in the top chamber drops below the top edge of the cup, the pressure inside the cup is given by Equation S1:

$$P = P_{\text{atm}} - \rho g h \tag{S1}$$

The liquid begins to boil when the pressure inside the cup, as described by the equation, approaches zero. This boiling prevents further transfer of the liquid, resulting in the state shown in Supplementary Figure S4. The video can be found in Supplementary files.

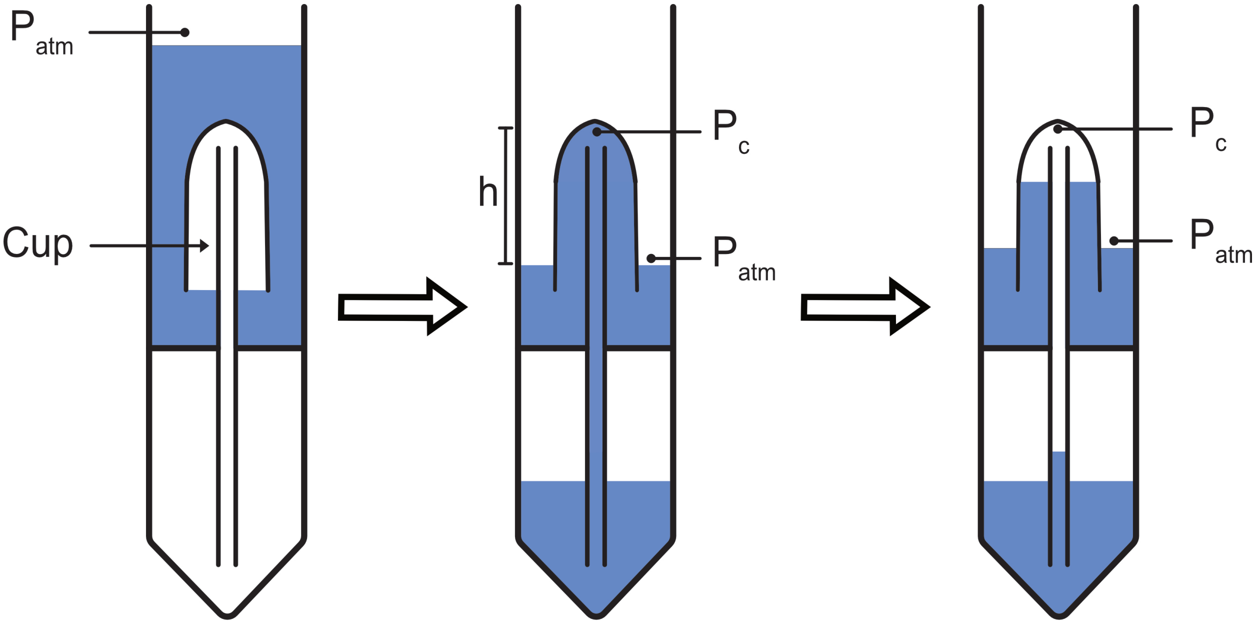

Figure S4: **Vacuum generation in the device by adjusting the cup's position.** During high-speed centrifugation (2500g), liquid transfer occurs via the siphon. When the liquid level in the top chamber drops below the top edge of the cup, the pressure inside the cup decreases, causing the liquid to boil and preventing further transfer.

Comparison with other separation methods

The key parameters for sample preparation methods from the blood for downstream detection include up-concentration factor, the minimum concentration of bacteria, blood processing capacity, throughput, and cell removal efficiency. These parameters are critical for the detection of bacterial presence, identification, and AST in the clinical management of sepsis and bloodstream infections (BSIs).

Up-Concentration Factor: The up-concentration factor is defined as the ratio of the bacterial concentration in the final aliquot to the initial concentration in the blood or blood culture. A higher up-concentration factor is advantageous for downstream detection, as the primary challenge in diagnosing sepsis or BSIs lies in the extremely low bacterial concentrations typically present in clinical samples. The developed device exhibits a significantly high up-concentration factor, making it well-suited for these applications.

Minimum Concentration of Bacteria: Given the low bacterial concentration in blood or blood culture during sepsis or BSI, it is crucial for any method or device to perform efficiently at such low levels. The developed device has been demonstrated to function effectively at bacterial concentrations as low as 10 CFU/mL, which is clinically relevant. This highlights its potential utility in addressing one of the most challenging aspects of sepsis diagnosis.

Throughput and Blood Volume Processing: Due to the low bacterial concentrations in clinical scenarios, processing large volumes of blood with high throughput is essential. The developed device demonstrates a superior throughput compared to many existing methods, as illustrated in the accompanying comparative data table. This capability enhances its applicability for routine clinical use.

Cell Removal Efficiency: In addition to bacterial concentration, the number of residual blood cells in the final aliquot significantly impacts the accuracy of bacterial detection. Residual blood cells can interfere with downstream detection processes, making blood cell removal efficiency a critical parameter. The developed device achieves a red blood cell (RBC) removal efficiency of 99.97%, which is exceptionally high and comparable to, if not better than, other existing methods.

In summary, the device demonstrates excellent performance across all critical parameters for sample preparation from blood or blood mixed with culture media, underscoring its potential for

enhancing the detection of sepsis and bloodstream infections in clinical settings.

Table S1: Key performance parameters of smart centrifugation and other separation methods.

	Up-concentration factor	Minimum concentration detected (CFU/ml)	Blood processed (ml)	Time (min)	Throughput (µl/min)	RBC removal efficiency
This device	3.20	14	3	40	75	99.97%
Smart centrifugation (24)	0.58	9	2.25	5	450	99.98%
Filter based centrifugation (26)	0.01 - 0.02	10	1	60	16.7	99.4%
Compact Disk (23)	0.67	10	7	1	7000	94%
Dextran sedimentation (25)	1.25-1.50	10-100	10	30	333	>90%
Elastoinertial Separation (30)	0.43	40	1	40	25	100%
Inertial lift forces (29)	0.02	$>10^5$	0.15	4	37.5	90%
Dielectrophoreis (32)	87.20	10^4	0.05	75	0.67	100%
SAW (31)	0.035	$4.4.10^7$	0.012	600	0.02	99.6%
Acoustophoresis (28)	0.25	5.10^8	1	50	20	99.99%
Magnetic Bead Separation (27)	4.00	1	5	60	83.3	>99.99%

Caption for Movie S1. Liquid motion in the device inside centrifuge. The video mentioned in the section ’Setup to film the liquid motion in the device inside the moving centrifuge’.

Caption for Movie S2. Vaccum generation inside the device. The video mentioned in the section ’Vaccum creation in the centrifugal device’.

Caption for Data S1. Raw data of the bacterial counts, blood cell rejection rates, and MALDI-TOF identification scores.
